# 723 Carbon Dioxide Laser Treatment Practices for Hypertrophic Burn Scars Amongst American Burn Association Burn Centers

**DOI:** 10.1093/jbcr/irac012.277

**Published:** 2022-03-23

**Authors:** Jon C Bruce, Travis S Dowdle, Kaylee B Schrader, John A Griswold

**Affiliations:** Texas Tech University Health Sciences Center, Lubbock, Texas; Texas Tech University Health Sciences Center School of Medicine, Lubbock, Texas; Texas Tech University Health Sciences Center School of Medicine, Amarillo, Texas; Texas Tech University Health Sciences Center, Lubbock, Texas

## Abstract

**Introduction:**

Scarring is a major outcome of severe burn wound healing. Severe scars often persist and diminish quality of life by disfigurement, pain, and pruritis. In the last decade, utilization of ablative fractional carbon dioxide (CO2) laser therapy has become a popular treatment modality for severe burn scars. Although the efficacy of CO2 lasers for the treatment of hypertrophic burn scars has been established via systematic reviews, there have been no attempts to query the 63 American Burn Association (ABA) centers across the United States regarding specific treatment parameters involving serious, sometimes high, total body surface area (TBSA) burns.

**Methods:**

Throughout October and November of 2020, a Qualtrics survey consisting of 14 questions was administered to burn surgeons practicing at all 63 ABA burn centers across the United States. Topics assessed were specific laser parameters utilized (5), treatment preferences (2), peri-operative follow-up (5), scar assessment practices (1), and TBSA treatment tolerance (1).

**Results:**

Exploratory, descriptive data was analyzed. Surgeons practicing at 27 of the 63 total ABA burn centers responded to the survey (43% response rate). Data elucidates the level of variance regarding current initial management of hypertrophic burn scars via CO2 laser treatment. Surgeons demonstrated variation in the level of TBSA treatment tolerance and pulse energy settings, respectively.

**Conclusions:**

Our findings show a substantial amount of variation in several aspects of CO2 laser hypertrophic scar revision between ABA centers across the country, including preoperative evaluation, laser settings, treatment regimen, and postoperative recommendations . Standardization of care when utilizing ablative fractional CO2 lasers should be further explored.

